# Supplementary material for: SkinDuoTM as a Targeted Probiotic Therapy: Shifts in Skin Microbiota and Clinical Outcomes in Acne Patients
Source: Int J Mol Sci. 2025 May 22;26(11):5000. doi: 10.3390/ijms26115000 (PMC12154497; doi:10.3390/ijms26115000)
Supplement: Supplementary file 1 [file ijms-26-05000-s001.zip › ijms-3633328-figure.pdf]

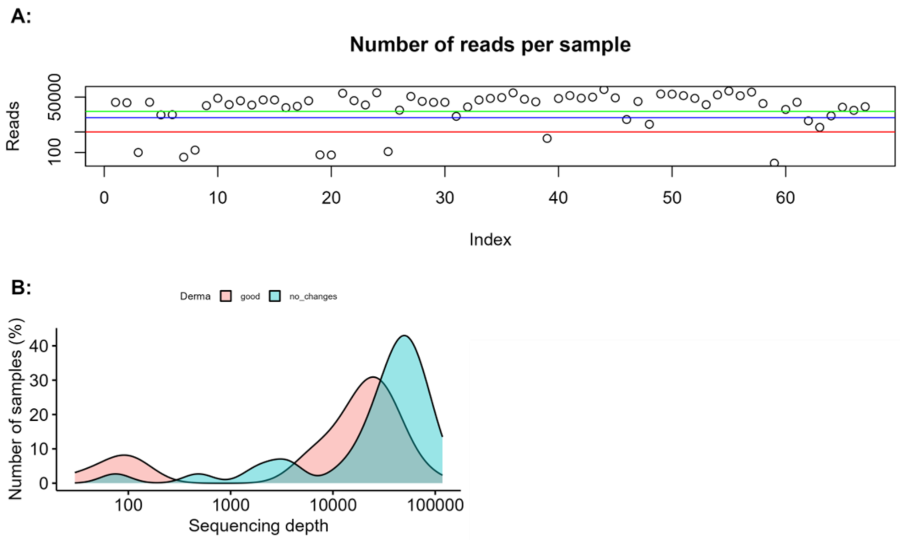

Figure S1. (A) Number of sequencings reads per sample. The red line indicates the threshold of 1,000 reads, below which samples were excluded from the analysis (n = 8; 03a, 09b, 19b, 21a, 24a_bis, 50b, 66b). The blue line represents the average number of reads across all samples (31,731 reads). Samples above the red threshold line were retained for further analyses, demonstrating that a sufficient number of reads were generated for the majority of samples. (B) Distribution of sequencing depth between the two groups, "good" responders (pink) and "no_changes" responders (blue). The density plot reveals a comparable read distribution across the groups, indicating no significant disparity in sequencing depth that could bias downstream analyses.
